# Application of Prognostic Models Based on Psoas Muscle Index, Stage, Pathological Grade, and Preoperative Carcinoembryonic Antigen Level in Stage II-III Colorectal Cancer Patients Undergoing Adjuvant Chemotherapy

**DOI:** 10.1155/2022/6851900

**Published:** 2022-02-02

**Authors:** Li Shan, Tian Li, Wenhao Gu, Yuting Gao, Erdong Zuo, Huizhu Qiu, Rong Li, Xu Cheng

**Affiliations:** ^1^Department of Hematology and Oncology, Taicang Hospital Affiliated to (Taicang No. 1 People's Hospital), Taicang, Suzhou 215400, China; ^2^School of Basic Medicine, Fourth Military Medical University, No. 169 Changle West Rd, Xi'an 710032, China; ^3^Department of Radiology, Taicang Hospital Affiliated to Soochow University (Taicang No. 1 People's Hospital), Taicang, Suzhou 215400, China

## Abstract

**Objective:**

To investigate the effect of sarcopenia on the prognosis of stage II-III colorectal cancer patients undergoing adjuvant chemotherapy.

**Methods:**

A total of 196 stage II-III colorectal cancer patients who received 8 cycles of postoperative chemotherapy were retrospectively analyzed. An abdominal CT acquired at 3-4 weeks after surgery was used to calculate the psoas muscle index. Subsequently, once gender-specific receiver operating characteristic curves were plotted and cut-off values of psoas muscle index were defined, the clinicopathological characteristics and the prognosis of patients with high and low values were compared. Lastly, prognostic models were established based on the independent prognostic factors of relapse-free survival and overall survival identified by COX analysis.

**Results:**

Based on the psoas muscle index, the prevalence of sarcopenia was 37.5% among 196 patients. This prevalence has significant correlation with patients' age and gender. However, it was not related to the AJCC stage, T stage, lymph node metastasis, pathological grade, grade III-IV myelosuppression, or preoperative carcinoembryonic antigen level. In addition, both the relapse-free and the overall survival of patients with low and high psoas muscle indexes were significantly different. COX analysis indicated that the psoas muscle index was an independent prognostic factor. Both the overall survival prognostic model based on patients' psoas muscle index, stage, pathological grade, and preoperative carcinoembryonic antigen level and the relapse-free survival prognostic model based on patients' psoas muscle index, pathological grade, and preoperative carcinoembryonic antigen level could accurately predict the prognosis of patients.

**Conclusion:**

For stage II-III colorectal cancer patients, the presence of sarcopenia before adjuvant chemotherapy would adversely affect their recurrence-free and overall survival. Prognostic models based on psoas muscle index, stage, pathological grade, and preoperative carcinoembryonic antigen level could accurately predict the prognosis in these patients.

## 1. Introduction

Colorectal cancer is one of the most common malignant digestive tract tumors in Europe and North America [1]. In China, colorectal cancer is the third most common cancer, ranking the third among all causes of cancer-related deaths in women and the fifth in men [2, 3]. Despite continuous progresses in treatment strategies, the survival rate of colorectal cancer remains poor due to late diagnosis, fast progression, and easy metastasis [4, 5]. The TNM (tumor, lymph node, and metastasis) staging system, which is widely used in the staging and prognostic prediction of patients with cancers including colorectal cancer, categorizes colorectal cancer patients into four different stages according to their TNM staging. Although theoretically, the prognosis of patients at the same stage should be similar, in clinical practice, vast differences are often observed. Therefore, it is necessary to identify new biological indicators to improve the accuracy of prognostic prediction [6, 7].

Sarcopenia is a syndrome characterized by the progressive and extensive loss of skeletal muscle mass and strength [8, 9]. According to the literature, sarcopenia has an effect on the postoperative complications and the long-term survival of patients with different cancers including gastric [10] esophageal [11] and colorectal cancer [12]. However, for stage II-III colorectal cancer patients receiving adjuvant chemotherapy, no studies have so far utilized CT before chemotherapy to determine the presence of sarcopenia as well as investigating the effect of sarcopenia on the prognosis.

Therefore, this study retrospectively analyzed the correlation between the incidence of sarcopenia and the clinicopathological characteristics, and the relapse-free survival (RFS), as well as the overall survival (OS) of 196 stage II-III colorectal cancer patients undergoing adjuvant chemotherapy. It was found that the psoas muscle index (PMI) was not only an effective indicator of the incidence of sarcopenia, but also one of the independent prognostic factors. Subsequently, for the first time, prognostic models based on patients' PMI after surgery were established for the prognostic prediction of these patients. Furthermore, because the parameters we utilized were from routine examinations during the baseline evaluation of colorectal cancer patients, these two models were economic, convenient, and accurate, making them suitable for further implementation.

## 2. Materials and Methods

Patients who were admitted to our institution between January 2011 and December 2018 were included in the study. This study was approved by the Ethics Committee of our institution (approval number: 2021-KY-155). All patients' diagnoses were confirmed by histopathology. In addition, enrolled patients underwent postoperative 8 cycles of 5-fluorouracil-based adjuvant chemotherapy in the Department of Oncology and were subsequently followed up. Prior written comprehensive informed consent for routine CT scan studies and treatment had been obtained from all patients. The TNM staging was performed according to the eighth edition of the American Joint Committee on Cancer (AJCC) colorectal cancer staging system (8th edition). Alternatively, the diagnosis of grade III-IV myelosuppression was based on WHO acute and subacute toxic effect grading criteria for anticancer drugs; that is, the patient was diagnosed to have grade III-IV myelosuppression if one of the following criteria was met: white blood cell count ≤1.9 × 10^9^/L, neutrophil count ≤0.9 × 10^9^/L, hemoglobin value ≤79 g/L, or platelet count ≤49 × 10^9^/L.

A routine and an enhanced abdominal scan was performed using Revolution CT scanner (GE Healthcare, Chicago, Illinois, United States) for all enrolled patients. The scan was acquired 3-4 weeks after surgery but before the start of systemic chemotherapy. Fasting for solids and liquids was required 8 h prior to the scan. Once the scan was completed, the cross-sectional area of bilateral psoas major muscles on the transverse plane of the lower edge of the third lumbar vertebral body on abdominal CT was measured by the same radiologist (Supplementary Figure 1). Next, the PMI was calculated as the sum of the area divided by the square of the patient's height, the unit of which was mm^2^/m^2^. The PMI is affected by patient gender owing to different body shapes of males and females. Consequently, gender-specific receiver operating characteristic (ROC) curves were plotted according to the recurrence status. Subsequently, once PMI cut-off values were defined based on Youden's index, corresponding indicators of patients in the high-PMI group and those in the low-PMI group were compared.

Observation indicators used in this study included patient age, gender, AJCC stage, T stage, N stage, pathological grade, presence of myelosuppression after chemotherapy, preoperative CEA level, RFS, OS, and PMI.

RFS and OS prognostic models were established based on the independent prognostic factors identified from multifactor COX analysis. Specific calculation formula is shown as follows:

(1) $$\begin{array}{c} \text{risk factor}=\sum_{i=1}^{n}{\text{Coef}}_{i}*x_{i}, \end{array}$$

where Coef_*i*_ is the risk factor and *x*_i_ is either the patient's stage (stage II = 0, stage III = 1), pathological grade (grade 1 = 1, grade 2 = 2, and grade 3 = 3), CEA value, or PMI (PMI high = 0, PMI low = 1). A prognostic nomogram model for patients' RFS and OS was then constructed according to the risk factors.

Patients were divided into a high-risk group and a low-risk group depending on whether their PMI was higher than the median of risk factors. Subsequently, the RFS and the OS of the two groups were compared by the Kaplan–Meier (KM) survival analysis, whereas the accuracy of the prognostic model in predicting patients' 1-year, 3-year, and 5-year RFS and OS was evaluated by the area under the ROC curve (AUC). Last, heatmap, risk score distribution map, and recurrence and survival state distribution map were adopted to determine whether the prognostic model could distinguish between high- and low-risk patients.

Statistical analysis: Other than the gender-specific ROC curves and the cut-off values, which were plotted in SPSS 23.0 software, all statistical analyses were conducted in R software (version 3.6.2). *P* < 0.05 was considered statistically significant. Correlations between the PMI and patients' clinical characteristics were assessed by logistic regression, whereas correlations between the PMI and other clinical parameters including RFS and OS were explored by univariate and multivariate COX regression analyses. The prognostic nomogram model was constructed using the RMS plugin of R software. Alternatively, correlations between the PMI and risk factors as well as RFS and OS were investigated using the KM estimator. The ROC analysis of risk factors was conducted using the survival ROC plugin of R software. Heatmap analysis was completed via the pheatmap plugin, data visualization was achieved via the Ggplot2 plugin of R software.

## 3. Results

Gender-specific ROC curves were first plotted according to the patient's recurrence state after surgery, so that the cut-off value could be determined. It was found that for male patients, the AUC and the cut-off value were 0.655 and 585.93 mm^2^/m^2^, respectively. Alternatively, for female patients, these values were 0.634 and 456.21 mm^2^/m^2^, respectively. Detailed results are shown in Supplementary Figure 2.

Of the 196 included patients, 109 were male and 87 were female. The median age was 64 years, and the average age was 62.2 ± 10.2 years (range 27–83). Seventy-five patients had stage II colorectal cancer, and the remaining 121 patients had stage III colorectal cancer. One patient's tumor was staged as T1, 10 as T2, 40 as T3, and 145 as T4. There were 121 patients with positive lymph nodes and 75 patients with negative ones. Based on differentiation, 9 cases were identified as well differentiated, 154 as moderately differentiated, and 33 as poorly differentiated. Forty-three cases developed grade II-IV myelosuppression. According to PMI measurements, 74 patients were divided into the low-PMI group, and the remaining 122 into the high-PMI group. Details are listed in Table 1.

Logistic analysis indicated that the PMI was correlated only with the patients' age and gender, but not with stage, T stage, lymph node metastasis, pathological grade, presence of grade III-IV myelosuppression, or preoperative CEA level. Detailed results are listed in Table 1.

**Figure 1:**
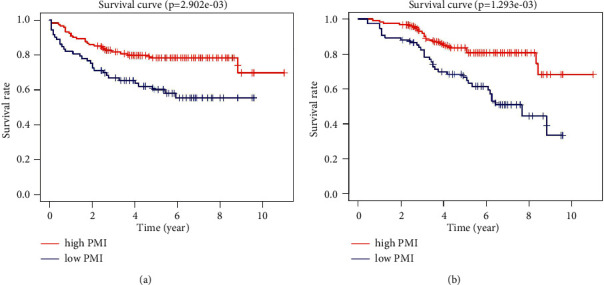
Low-PMI colorectal cancer patients is associated with poor RFS and OS. (a) RFS; (b) OS.

KM analysis suggested that both the RFS and the OS of patients with a low PMI were significantly poorer than those of patients with a high PMI (*P*=0.003 and 0.001, respectively). More specifically, the 5-year RFS and OS of low-PMI patients were merely 60.2% and 63.4%, whereas for high-PMI patients, these were 78.5% and 80.7%, respectively, as shown in Figures 1(a) and 1(b).

**Figure 2:**
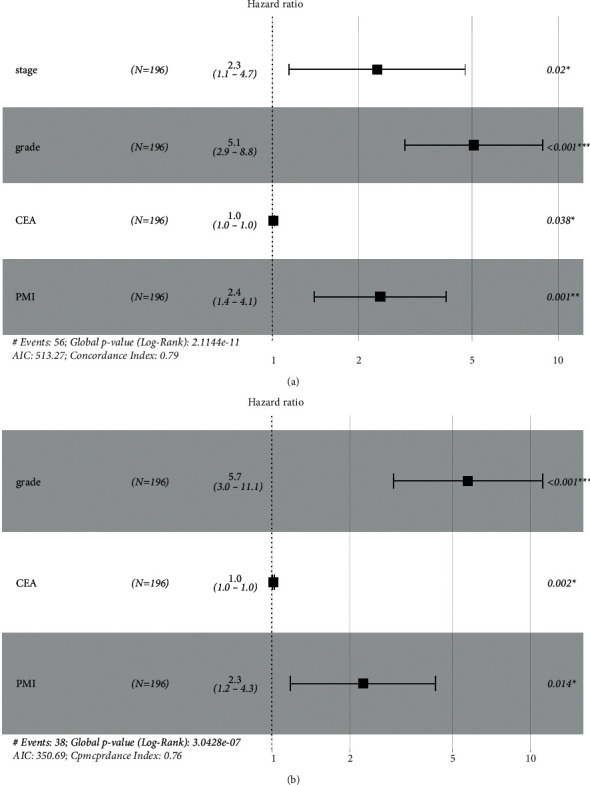
Forest plot of patients' RFS and OS from multivariate COX regression analysis. (a) RFS; (b) OS.

Univariate COX regression analysis showed that patients' RFS and OS were related to multiple clinicopathological characteristics, including the patient's stage, lymph node metastasis, pathological grade, presence of grade III-IV myelosuppression after chemotherapy, preoperative CEA level, and PMI. In contrast, multivariate COX regression analysis indicated that the RFS was correlated with the patient's stage, pathological grade, preoperative CEA level, and PMI, whereas the OS was correlated with the pathological grade, preoperative CEA level, and PMI. In addition, PMI was identified as an independent prognostic factor of patients' RFS and OS. Detailed results are shown in Tables 2 and 3 and Figures 2(a) and 2(b).

**Figure 3:**
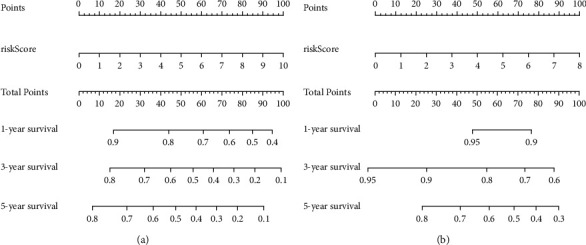
Prognostic model nomograms. (a) RFS; (b) OS.

An RFS prognostic model was established based on patients' PMI, stage, pathological grade, and preoperative CEA level. The risk score = PMI *∗* 0.868 + stage *∗* 0.843 + pathological grade *∗* 1.623 +preoperative CEA level *∗* 0.009. Similarly, an OS prognostic model was constructed based on patients' PMI, pathological grade, and preoperative CEA level. The risk score = PMI  *∗* 0.812 + pathological grade *∗* 1.747 + preoperative CEA level *∗* 0.013. As shown in Figures 3(a) and 3(b), a prognostic nomogram model for patients' RFS and OS was then constructed according to the above risk factors.

**Figure 4:**
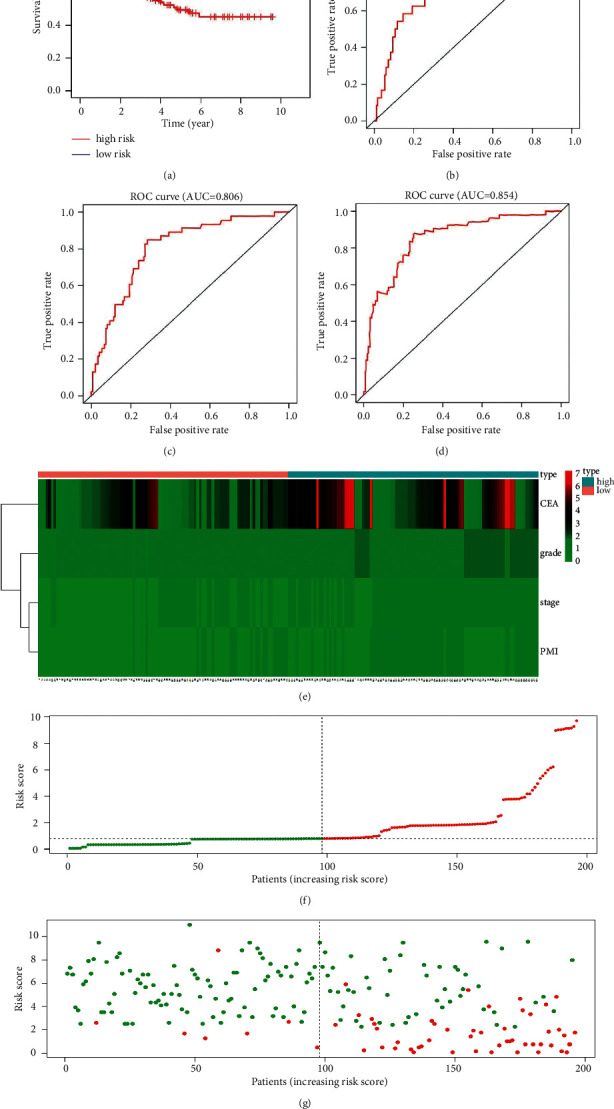
Evaluation of the prognostic in predicting patients' RFS. (a) KM analysis; (b) 1-year ROC; (c) 3-year ROC; (d) 5-year ROC; (e): heatmap; (f) risk score distribution map; (g) recurrence state distribution map.

It was found that the RFS of high-risk patients was substantially lower than that of low-risk patients (*P* = 1.1*E* − 11). The 5-year RFS of the two groups was 49.4% and 93.7%. The AUC of the 1-year, 3-year, and 5-year ROC curves was 0.840, 0.806, and 0.854, respectively as shown in Figures 4(a)–4(d). Heatmap, risk score, and recurrence state distribution map all indicated that the prognostic model could accurately distinguish the RFS status of high-risk patients from that of low-risk patients, as shown in Figures 4(e)–4(g).

**Figure 5:**
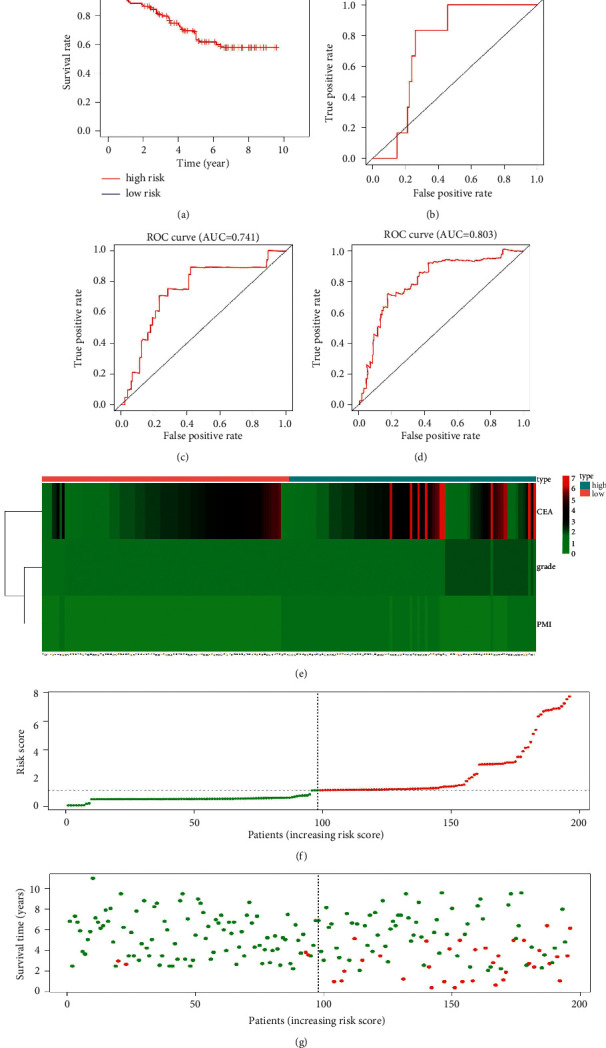
Evaluation of the prognostic in predicting patients' OS. (a) KM analysis; (b) 1-year ROC; (c) 3-year ROC; (d) 5-year ROC; (e) heatmap; (f) risk score distribution map; (g) survival state distribution map.

It was found that the OS of high-risk patients was substantially lower than that of low-risk patients (*P* = 1.1*E* − 11). The 5-year OS of the two groups of patients was 63.4% and 95.0%. The AUC of the 1-year, 3-year, and 5-year ROC curves was 0.744, 0.741, and 0.803, respectively, as shown in Figures 5(a)–5(d). Heatmap, risk score, and survival state distribution map all indicated that the prognostic model could accurately distinguish the OS status of high-risk patients from that of low-risk patients, as shown in Figures 5(e)–5(g).

## 4. Discussion

According to the consensus of the European Working Group on Sarcopenia in Older People in 2010, sarcopenia can be categorized as primary or secondary depending on the cause [13]. Primary sarcopenia is defined as muscle loss that is only related to age and does not have any obvious cause. In contrast, secondary sarcopenia is often caused by one or more obvious reasons such as inflammatory diseases, malignant tumors, and malnutrition [14–16].

Accurate diagnosis of sarcopenia currently requires the determination of three parameters: muscle strength, muscle mass, and physical fitness [17, 18], although it remains controversial how these three indicators should be applied to the diagnosis of the disease. At present, the most used indicator is the muscle mass. The European Working Group on Sarcopenia in Older People has described CT and MRI as the gold standards for estimating muscle mass [19]. CT scan has now been adopted as a routine examination during the diagnosis, staging, and monitoring of cancer patients, making it a suitable method of assessing muscle mass. However, the measurement of systemic skeletal muscle is not only extremely complicated, but also inconvenient for clinical practice. Although the study has suggested that the amount of skeletal muscle in the third lumbar vertebral plane is directly proportional to the amount of skeletal muscle in the entire body [19], the measurement of the former is equally complicated and prone to error. Recently, another study has indicated that the PMI is significantly related to the amount of skeletal muscle among the Asian population [20]. Therefore, in our study, the PMI was adopted as the indicator of muscle mass, which was subsequently utilized to plot gender-specific ROC curves. In addition, PMI cut-off values were determined according to Youden's index as the standard to diagnose sarcopenia, and correlations between the incidence of sarcopenia and the clinicopathological characteristics as well as the prognosis of stage II-III colorectal cancer patients undergoing adjuvant chemotherapy were investigated.

Research by Lieffers et al. [21] found that the overall prevalence of sarcopenia in 234 stage II-IV colorectal cancer patients was 38.9%. Alternatively, Miyamoto et al. [22] reported that the incidence of sarcopenia in 220 stage I-III colorectal cancer patients was 25%. Similarly, our study discovered that the prevalence of sarcopenia in 196 stage II-III colorectal cancer patients undergoing adjuvant chemotherapy was 37.5%, a ratio that is of significance. In addition, we found that the incidence of a low PMI in patients aged over 60 years and in patients aged less than 60 years was 42.01% and 24.68%, respectively, whereas the incidence of a low PMI in female and male patients was 50.57% and 20.78%, respectively. In both comparisons, the difference was statistically significant, which was consistent with the results from relevant research on the correlation between the prevalence of sarcopenia and the patient's age and gender [23].

As was previously mentioned, an important cause of sarcopenia is cancer. Therefore, theoretically, it is expected that patients' tumor stage should be related to the incidence of sarcopenia. A study by Zhuang et al. revealed the relationship between sarcopenia and the stage, T stage, and lymph node metastasis of gastric cancer patients [24]. In contrast, another study by McSorley et al. [25] reported that there was no correlation between the incidence of sarcopenia and the TNM stage of colorectal cancer patients. This is consistent with our findings that the incidence of low PMI is irrelevant to the patient's stage, T stage, N stage, or pathological grade. This result suggested that the correlation between sarcopenia and the clinical characteristics of stage II-III colorectal cancer patients required further investigation. Even though no correlation between patients' T stage and sarcopenia was found, the decline in the PMI showed a clear upward trend with increasing N stage, especially between N0, N1, and N2 (N0 vs. N1 vs. N2: 32.00% vs. 32.81% vs. 50.88%, *P*=0.064). Therefore, it was concluded that compared with the local size and local invasion of the tumor, the lymph node metastasis status could exhibit a greater impact on the incidence of sarcopenia. Alternatively, while the action mechanism of sarcopenia on the side effects of chemotherapy has not been clarified, multiple studies have reported that sarcopenia increased the risk of chemotherapy-related grade III-IV toxicity among colon cancer patients [12, 26]. However, in our study, no correlation between sarcopenia and grade III-IV myelosuppression was observed. This was likely because most stage II and a small number of stage III patients included in this study only received capecitabine single-agent chemotherapy. Therefore, the fact that only 43 patients developed grade III-IV myelosuppression was likely a result of the weak intensity of chemotherapy. In addition, the small sample size could also contribute to a possible bias, and no grade III-IV side effects other than myelosuppression were considered, which could also contribute to a possible bias.

Sarcopenia is a response to increased tumor biological activity and metabolism, the latter of which first causes a severe systemic inflammatory response and ultimately leads to muscle loss [27]. Several recent studies have indicated that a systemic inflammatory response is directly related to the prognosis of multiple malignant tumors [28]. For example, overall survival was significantly associated with increased neutrophil-to-lymphocyte and decreased lymphocyte-to-monocyte ratio in patients with rectal cancer. [29] This result suggested that the infiltration of inflammatory cells into the tumor is a factor of poor prognosis for patients with rectal cancer. Furthermore, since it has been reported that the actin secreted by muscle cells can inhibit the growth of tumor cells [29], sarcopenia can increase the risk of tumor recurrence and compromise the patient's OS by incurring actin damage. In a retrospective analysis of 220 stage I-III colorectal cancer patients who received radical resection, Miyamoto et al. found that patients with sarcopenia had considerably less RFS and OS. Another meta-analysis of 12 studies including 5,337 nonmetastatic colorectal cancer patients also reported that sarcopenia was a negative factor for patient's survival outcome. However, up until now, there have been no studies that either utilized CT to determine the presence of sarcopenia before chemotherapy among stage II-III colorectal cancer patients receiving adjuvant chemotherapy or investigated the effect of sarcopenia on the prognosis of the same patient group. Therefore, this study retrospectively analyzed the relationship between the incidence of sarcopenia and the RFS as well as the OS of 196 patients. The results showed that the 5-year RFS and OS for patients with a low PMI were merely 60.2% and 63.4%, respectively. However, for patients with a high PMI, these were 78.5% and 80.7%, which were significantly higher (*P*=0.003 and 0.001, respectively). This finding suggested that sarcopenia affected the RFS and OS of stage II-III colorectal cancer patients receiving postoperative adjuvant chemotherapy. In addition, Wang et al. discovered that the incidence of sarcopenia before surgery is an independent prognostic factor for colorectal cancer patients [30]. Similarly, in this study, univariate COX regression analysis found that patients' RFS and OS were related to their tumor stage, lymph node metastasis, pathological grade, presence of grade III-IV myelosuppression, preoperative CEA level, and PMI. Alternatively, multivariate COX regression analysis suggested that patients' RFS was correlated with their stage, pathological grade, preoperative CEA level, and PMI, whereas their OS was correlated with their pathological grade, preoperative CEA level, and PMI. Based on these results, it was concluded that the PMI was an independent prognostic factor for the RS and the OS of stage II-III colorectal cancer patients undergoing adjuvant chemotherapy.

The study also established an RFS prognostic model based on patients' PMI, stage, pathological grade, and preoperative CEA level, as well as an OS prognostic model based on patients' PMI, pathological grade, and preoperative CEA level. Both models were subsequently verified by KM analysis, ROC analysis, heatmap, risk score distribution map, and recurrence status distribution map. The results indicated that the prognostic models could accurately predict patients' 1-, 3-, and 5-year RFS and OS as well as distinguishing between low- and high-risk patients.

In conclusion, approximately one-third of stage II-III colorectal cancer patients undergoing postoperative adjuvant chemotherapy could develop sarcopenia, the incidence of which was an independent prognostic factor of patients' RFS and OS. The use of the PMI in determining the presence of sarcopenia is both convenient and economic. Therefore, for colorectal cancer patients who have undergone radical surgery, their PMIs should be measured prior to the start of adjuvant chemotherapy to predict prognosis. For patients with a low PMI, individualized interventions such as nutritional support can be considered to increase muscle quantity and quality and consequently improve prognosis. Prognostic models established in this study based on the PMI, stage, pathological grade, and preoperative CEA level can accurately predict the prognosis of stage II-III colorectal cancer patients undergoing postoperative adjuvant chemotherapy and therefore should be implemented in the future.

This study had several limitations. First, it was a retrospective study conducted in a single center. Second, some prognostic factors for colon cancer patients, such as buddings and lymph node ratio, were not included in our COX regression analysis [31, 32]. Third, there are other techniques widely used to assess muscle mass, such as magnetic resonance imaging and bioelectric impedance analysis [18], which we were unable to perform because this study was a retrospective analysis and it was possible to compare the prevalence of muscle loss between those techniques and PMI. Hence, comprehensive studies with multicenters and multitechniques are warranted in the future.

## Figures and Tables

**Table 1:** Correlation between patients' clinicopathological characteristics and PMI.

Clinical characteristics		Total no.	PMI		Ratio of low PMI (%)	Odds ratio in PMI	*P*
			High	Low			
Age	>60 years old	119	64	55	42.01	1.031 (1.001–1.063)	0.045
	≤60 years old	77	58	19	24.68		

Gender	Male	109	79	30	27.78	0.225 (0.225–0.735)	0.003
	Female	87	43	44	50.57		

Stage	II	75	51	24	32.00	1.361 (0.746–2.485)	0.315
	III	121	71	50	41.32		

T stage	T1	1	0	1	100.00	0.823 (0.509–1.329)	0.425
	T2	10	3	7	70.00		
	T3	40	30	10	25.00		
	T4	145	89	56	38.62		

Lymph node	N0	75	51	24	32.00	1.395 (0.980–1.986)	0.064
	N1	64	43	21	32.81		
	N2	57	28	29	50.88		

Pathological grade	G1	9	7	2	22.22	0.797 (0.414–1.534)	0.497
	G2	154	91	63	40.91		
	G3	33	24	9	27.27		

CEA	High	73	46	27	36.99	0.998 (0.985–1.101)	0.751
	Normal	123	76	47	38.21		

Myelosuppression	Grade III-IV	43	25	18	41.86	1.247 (0.626–2.485)	0.530

**Table 2:** COX regression analysis results on correlations between patients' PMI, clinicopathological characteristics, and RFS.

Parameter	Univariate COX analysis			Multivariate COX analysis			
	HR	95% CI	*P*	Coef	HR	95% CI	*P*
Age	1.020	0.992–1.048	0.162	—	0.997	0.969–1.025	0.828
Gender	0.794	0.470–1.342	0.389	—	0.969	0.543–1.726	0.914
Stage	3.395	1.712–6.734	0.001	0.843	2.324	1.143–4.727	0.020
T	1.071	0.676–1.697	0.769		0.994	0.636–1.554	0.981
N	2.046	1.478–2.832	1.60*E* − 05	—	1.054	0.571–1.947	0.866
Pathological grade	5.082	3.012–8.575	1.11*E* − 09	1.623	5.066	2.909–8.821	9.81*E* − 09
Grade III-IV myelosuppression	2.859	1.675–4.880	1.17*E* − 04	—	1.403	0.772–2.548	0.267
CEA	1.009	1.000–1.016	0.041	0.009	1.009	1.000–1.017	0.038
PMI	2.315	1.366–3.923	0.002	0.868	2.382	1.398–4.058	0.001

**Table 3:** COX regression analysis results on correlations between patients' PMI, clinicopathological characteristics, and OS.

Parameter	Univariate COX analysis			Multivariate COX analysis			
	HR	95% CI	*P*	Coef	HR	95% CI	*P*
Age	1.041	1.005–1.078	0.026	—	1.023	0.984–1.063	0.253
Gender	1.003	0.529–1.902	0.993	—	1.289	0.641–2.590	0.476
Stage	3.279	1.442–7.455	0.005	—	1.163	0.257–5.262	0.845
T	1.221	0.669–2.230	0.516	—	1.001	0.559–1.795	0.996
N	2.043	1.374–3.036	4.14*E* − 04	—	1.297	0.591–2.846	0.516
Pathological grade	4.607	2.461–8.623	1.79*E* − 06	1.747	5.737	2.956–11.135	2.42*E* − 07
Grade III-IV myelosuppression	3.229	1.703–6.123	3.29*E* − 04	—	1.885	0.921–3.859	0.083
CEA	1.011	1.003–1.020	0.010	0.013	1.013	1.005–1.022	0.002
PMI	2.110	1.113–4.000	0.022	0.812	2.252	1.179–4.302	0.014

## Data Availability

The raw data are available in Supplementary Materials. More information can be accessed from the corresponding author.

## Abbreviations

TNM: Staging, tumor, lymph node, and metastasis staging
RFS: Relapse-free survival
OS: Overall survival
PMI: Psoas muscle index
CEA: Carcinoembryonic antigen
AJCC: American Joint Committee on Cancer
ROC: Receiver operating characteristic
KM: Kaplan–Meier.

## Supplementary Materials

Supplementary Materials
*Supplementary Figure 1.* Assessment of the psoas muscle index (PMI). PMI was calculated as the sum of cross-sectional area of bilateral psoas major muscles in the lower edge of the third lumbar vertebral body (L3) and divided by the square of the patient's height. (A) Right sectional area of psoas major muscles; (B) left sectional area of psoas major muscles. *Supplementary Figure 2.* Gender-specific ROC curves. (A) Male patients; (B) female patients.
