# Supplementary figures and images for: Application of Prognostic Models Based on Psoas Muscle Index, Stage, Pathological Grade, and Preoperative Carcinoembryonic Antigen Level in Stage II-III Colorectal Cancer Patients Undergoing Adjuvant Chemotherapy
Source: J Oncol. 2022 Feb 2;2022:6851900. doi: 10.1155/2022/6851900 (PMC8828329; doi:10.1155/2022/6851900)

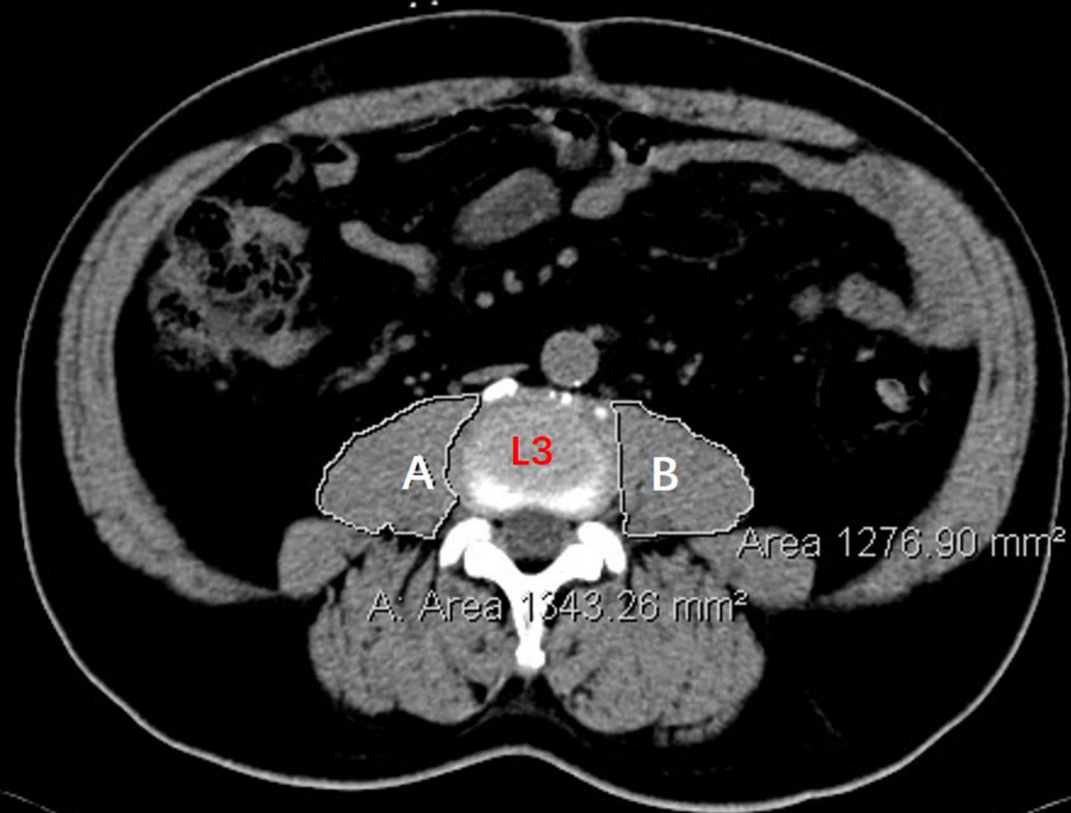

Supplement: Supplementary Materials — Supplementary Figure 1. Assessment of the psoas muscle index (PMI). PMI was calculated as the sum of cross-sectional area of bilateral psoas major muscles in the lower edge of the third lumbar vertebral body (L3) and divided by the square of the patient's height. (A) Right sectional area of psoas major muscles; (B) left sectional area of psoas major muscles. Supplementary Figure 2. Gender-specific ROC curves. (A) Male patients; (B) female patients. [file 6851900.f1.zip › 6851900.f1/Supplementary figure 1.pdf]

A

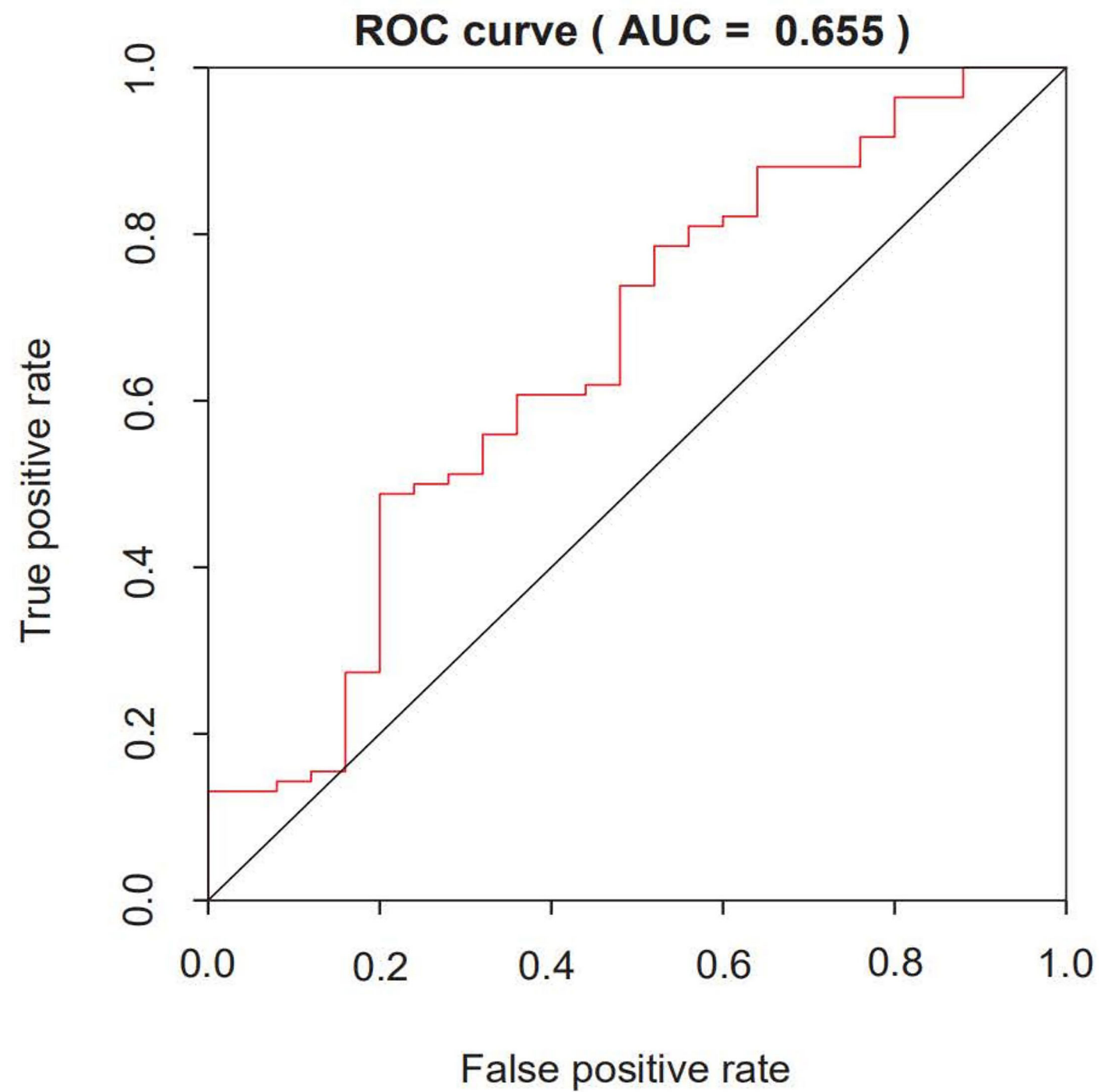

B

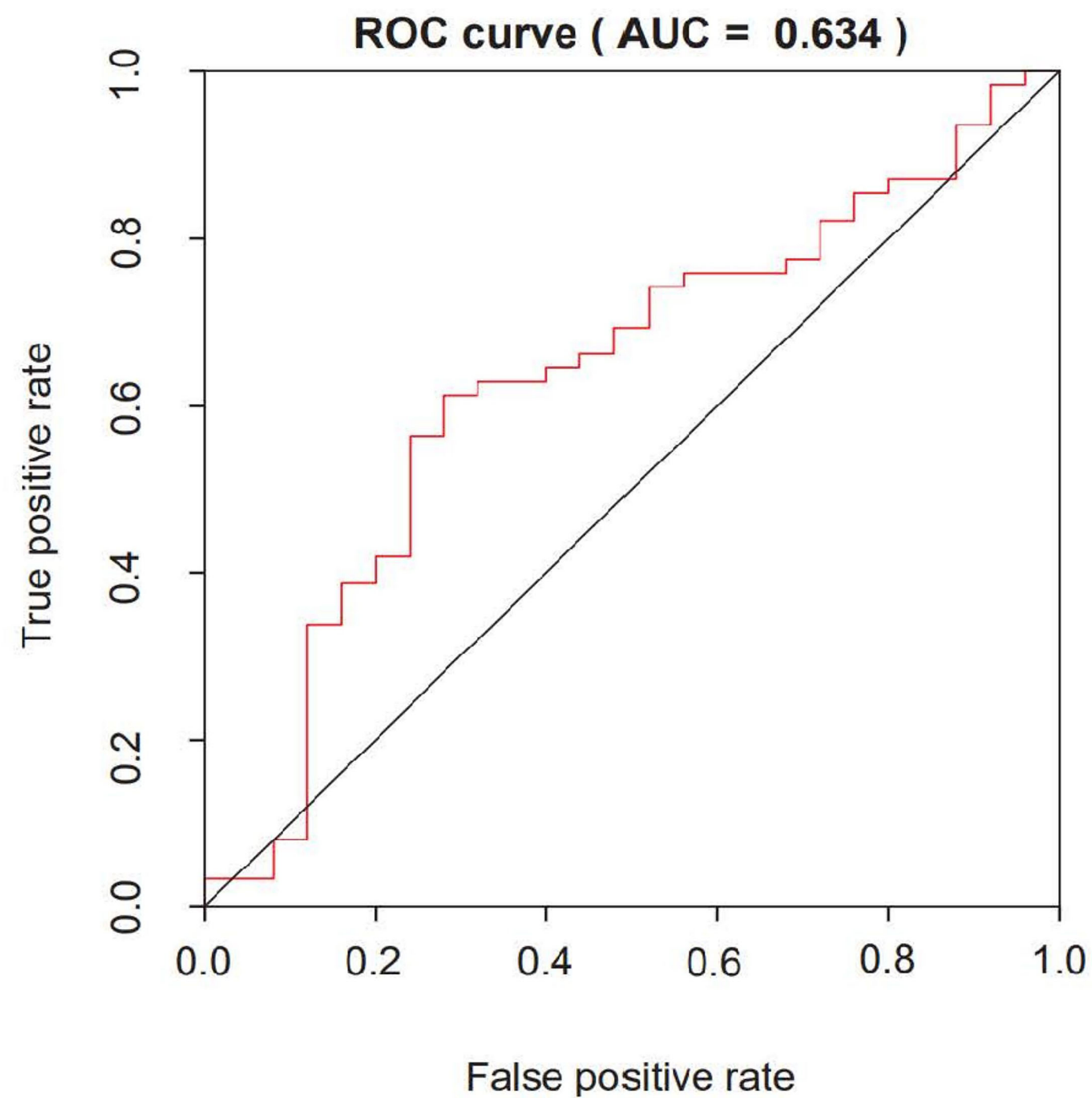

Supplement: Supplementary Materials — Supplementary Figure 1. Assessment of the psoas muscle index (PMI). PMI was calculated as the sum of cross-sectional area of bilateral psoas major muscles in the lower edge of the third lumbar vertebral body (L3) and divided by the square of the patient's height. (A) Right sectional area of psoas major muscles; (B) left sectional area of psoas major muscles. Supplementary Figure 2. Gender-specific ROC curves. (A) Male patients; (B) female patients. [file 6851900.f1.zip › 6851900.f1/Supplementary figure 2.pdf]
